# Dickkopf-1 induces angiogenesis via VEGF receptor 2 regulation independent of the Wnt signaling pathway

**DOI:** 10.18632/oncotarget.19769

**Published:** 2017-08-01

**Authors:** Sung Hoon Choi, Hyemi Kim, Hyun Gyu Lee, Beom Kyung Kim, Jun Yong Park, Do Young Kim, Sang Hoon Ahn, Kwang-Hyub Han, Seung Up Kim

**Affiliations:** ^1^ Division of Bioconvergence Analysis, Drug and Disease Target Group, Korea Basic Science Institute, Daejeon, Korea; ^2^ Department of Microbiology and Immunology, Institute of Gastroenterology, Yonsei University College of Medicine, Seoul, Korea; ^3^ Brain Korea 21 Plus Project for Medical Sciences, Institute of Gastroenterology, Yonsei University College of Medicine, Seoul, Korea; ^4^ Department of Internal Medicine, Institute of Gastroenterology, Yonsei University College of Medicine, Seoul, Korea; ^5^ Liver Cirrhosis Clinical Research Center, Seoul, Korea

**Keywords:** dickkopf-1, angiogenesis, HUVEC, Hepatocellular carcinoma, vascular endothelial growth factor receptor

## Abstract

Tumor angiogenesis is essential for invasive tumor growth and metastasis. Dickkopf-1 (DKK-1), an antagonist of Wnt signaling, participates in tumor development and progression. We evaluated whether DKK-1 stimulation induces angiogenesis and the endothelial–mesenchymal transition (EnMT).

Human umbilical vein endothelial cells (HUVECs) were stimulated with recombinant DKK-1 (rDDK-1) or conditioned medium from a culture of DKK-1-transfected 293 cells. Following stimulation, the expression levels of angiogenesis-related factors and EnMT related markers were determined by immunoblot assays. In addition, the effects of exogenous DKK-1 on angiogenesis and EnMT were assessed by tube-formation, cell invasion, and wound-healing assays.

Human hepatoma cells, such as Hep3B and Huh-7, showed high levels of DKK-1 expression, whereas 293 cells and HUVECs showed little or no DKK-1 expression. Increased endothelial cell tube formation and invasiveness were observed in HUVECs treated with concentrated conditioned medium from DKK-1-overexpressing 293 cells or rDKK-1. DKK-1-stimulated HUVECs also exhibited increased motility in wound-healing assays. Furthermore, the expression levels of angiogenesis-related factors, including vascular endothelial growth factor receptor 2 and vascular endothelial-cadherin, were increased in DKK-1-stimulated HUVECs. The expression of EnMT markers, such as vimentin and Twist, was also increased in DKK-1-stimulated HUVECs. However, no significant change in β-catenin or GSK3β expression was observed.

Our *in vitro* data suggest that DKK-1 can enhance angiogenesis and EnMT by HUVECs independent of the Wnt signaling pathway. Modulation of DKK-1 expression may facilitate development of novel strategies to control tumor angiogenesis and metastasis.

## INTRODUCTION

Dickkopf-1 (DKK-1) is a potent antagonist of Wnt/β-catenin signaling [1]. DKK-1 acts as an inhibitory ligand of the low-density lipoprotein receptor-related protein 5/6 co-receptors and subsequently blocks their interaction with Wnt, resulting in β-catenin degradation [2]. This inhibitory role of DKK-1 in Wnt/β-catenin signaling is supported by the downregulation of DKK-1 in human colon cancers and the correlation between high DKK-1 expression and favorable responses to chemotherapy in brain tumors [3]. In contrast, DKK-1 overexpression has been reported in various tumors, including human hepatoblastomas, Wilms’ tumors, multiple myelomas, and hormone-resistant breast cancers [4–6]. In addition, DKK-1 is highly expressed in hepatocellular carcinoma (HCC) [7], which is significantly correlated with poorer pathologic grade and postoperative outcomes [8, 9].

To date, it has been demonstrated that DKK-1 promotes angiogenesis during development, tumorigenesis, and inflammation [10]. DKK-1 enhances the angiogenic properties of human endothelial colony-forming cells, and increases tumoral angiogenesis in breast cancer [11]. In osteoarthritic knee joints, DKK-1 was associated with angiogenesis and cartilage matrix proteinase secretion [12]. In addition, Tung et al. reported that a DKK-1expressing HCC cell line showed an increased microvessel density around tumors [13], while another study showed that DKK-1–mediated endothelial cell activation led to increased vascular density and vessel diameter in rats [14]. These findings indicate that DKK-1 plays a role in microvascular remodeling and tumor angiogenesis activation, which may account for its promotion of cancer growth *in vivo*.

However, the mechanism underlying the role of DKK-1 in angiogenesis remains to be elucidated. In the present study, DKK-1 expression was determined in mouse HCC tissue and human HCC cell lines. In addition, the effects of DKK-1 on angiogenesis and endothelial–mesenchymal transition (EnMT) were investigated.

## RESULTS

### DKK-1 expression in HCC

**Figure 1:**
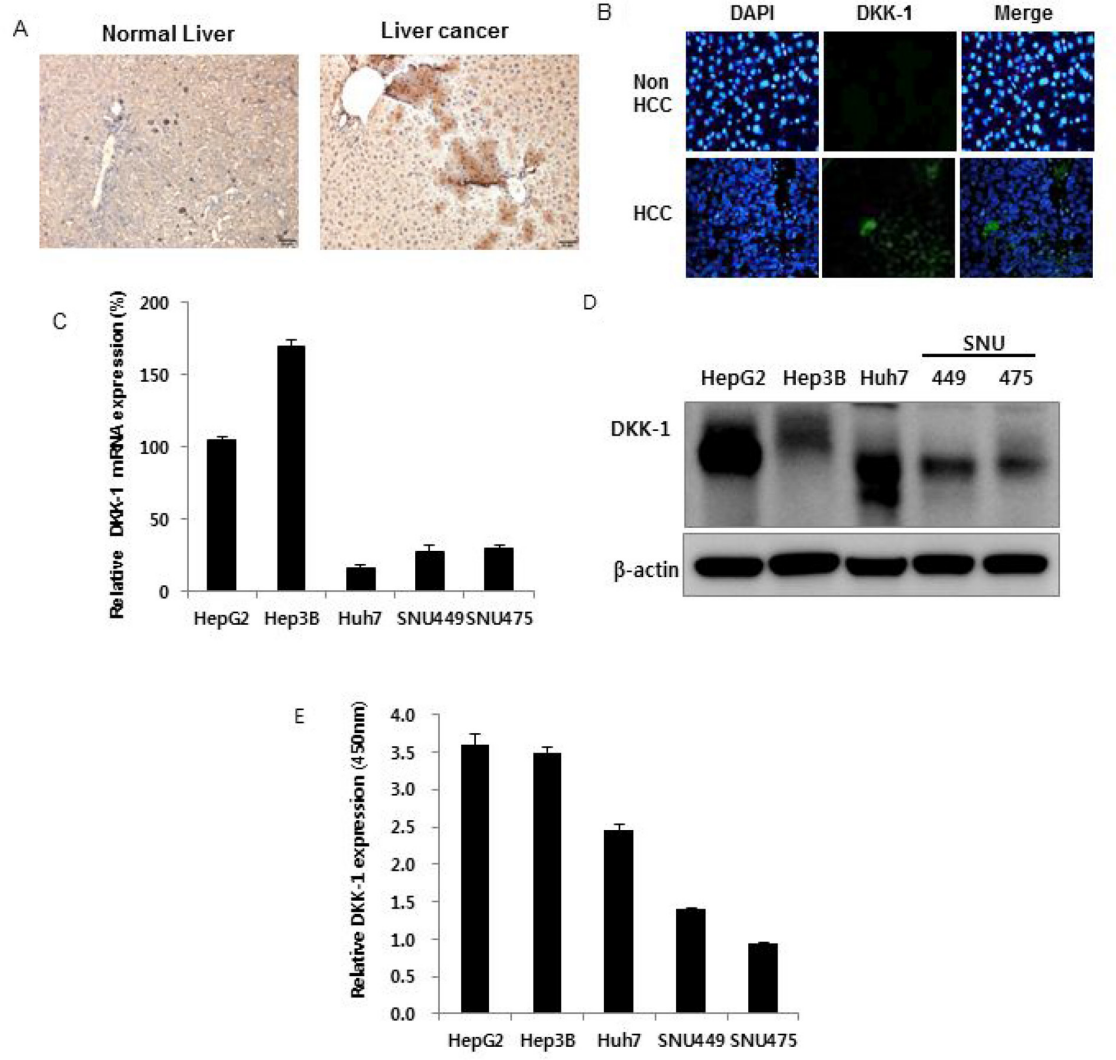
Dickkopf-1 (DKK-1) expression in hepatocellular carcinoma (HCC) DKK-1 expression in c-myc-overexpressing HCC mice tissue (**A**) and xenograft mice using Hep3B cells (**B**). DKK-1 mRNA (**C**) protein (**D**) and secreted protein (**E**) levels were high in HepG2, Hep3B, and Huh7 cells, but lower in SNU 449 and SNU 475 cells.

DKK-1 expression was high in c-myc-overexpressing HCC mice tissue and in a Xenograft mouse model using the Hep3B cell line (Figures 1A and 1B) [15, 16], compared to the controls. In addition, DKK-1 was expressed in several human HCC cell lines. DKK-1 mRNA, DKK-1 protein, and secreted DKK-1 protein levels (Figure 1C–1E) were high in HepG2, Hep3B, and Huh7 cells but lower in SNU 449 and SNU 475 cells. In contrast, DKK-1 was not expressed in human umbilical vein endothelial cells (HUVECs) or 293 cells (Supplementary Figure 1). All these support that DKK-1 expression is related to HCC development.

### DKK-1 increases motility, invasion, and tube formation

**Figure 2:**
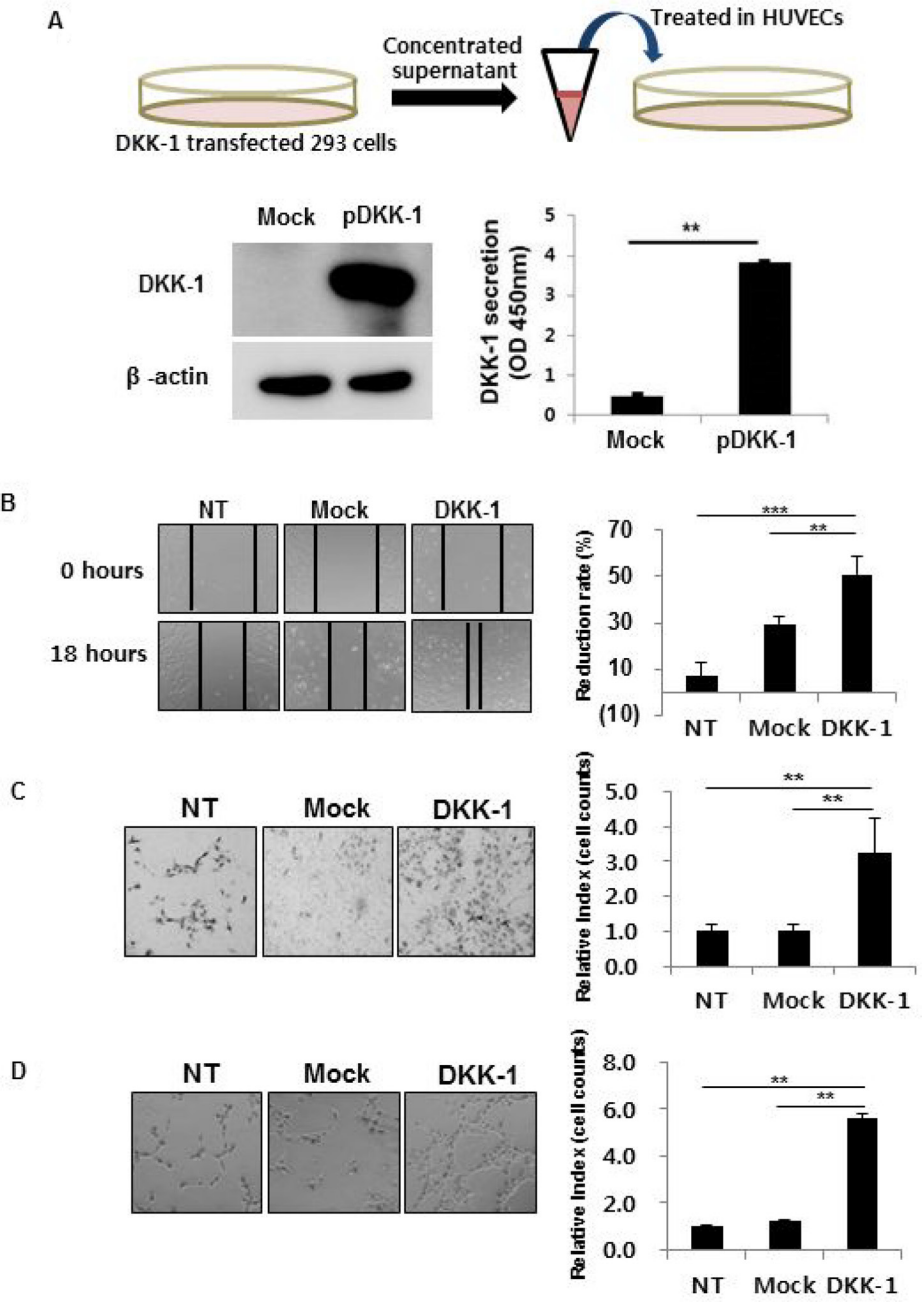
DKK-1 increases the motility, invasion, and tube formation of human umbilical vein endothelial cells (HUVECs) DKK-1 conditioned medium was manufactured by transfecting DKK-1 into 293 cells and confirmed by Western blot and ELISA (**A**). HUVECs cultured in DKK-1-conditioned medium exhibited increased motility (**B**), invasion (**C**), and tube formation (**D**) compared to the controls. NT, non-treated.

The effect on angiogenesis of DKK-1 stimulation was investigated. Concentrated conditioned medium of DKK-1-expressing 293 cells was first produced (Figure 2A). A high concentration of secreted DKK-1 protein (pDKK-1) was confirmed by Western blot and ELISA (Figure 2A).

DKK-1 stimulation increased the motility, invasion, and tube formation of HUVECs compared to controls, suggesting increased angiogenetic potential (Figure 2B–2D). Treatment with recombinant DKK-1 (rDKK-1) resulted in concentration-dependent increases in motility, invasion, and tube formation by HUVECs (Supplementary Figure 2). In contrast, DKK-1 did not affect HUVEC proliferation, suggesting that cell proliferation did not influence the results of the motility, invasion, and tube formation assays (Supplementary Figure 3).

### DKK-1 increases EnMT marker expression

**Figure 3:**
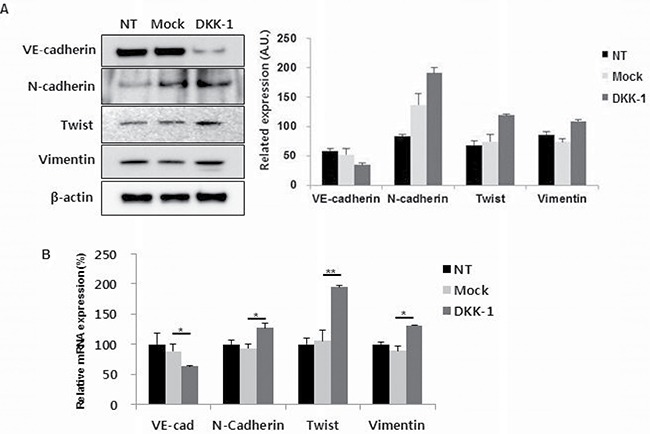
DKK-1 increases the expression of endothelial–mesenchymal transition (EnMT) markers in HUVECs DKK-1 stimulation resulted in increased N-cadherin, twist, and vimentin expression, and decreased VE-cadherin expression, compared to the controls by western blot (**A**) and mRNA level (**B**). NT, non-treated.

To investigate the influence of DKK-1 on EnMT, HUVECs were stimulated by DKK-1. DKK-1 stimulation led to increased N-cadherin, Twist and vimentin expression, and decreased vascular-endothelial (VE)-cadherin expression, compared to the controls (Figure 3A and 3B). rDKK-1 exerted similar effects, in a concentration-dependent manner (Supplementary Figure 4).

### Effect of DKK-1 on gene expression profiles

**Figure 4:**
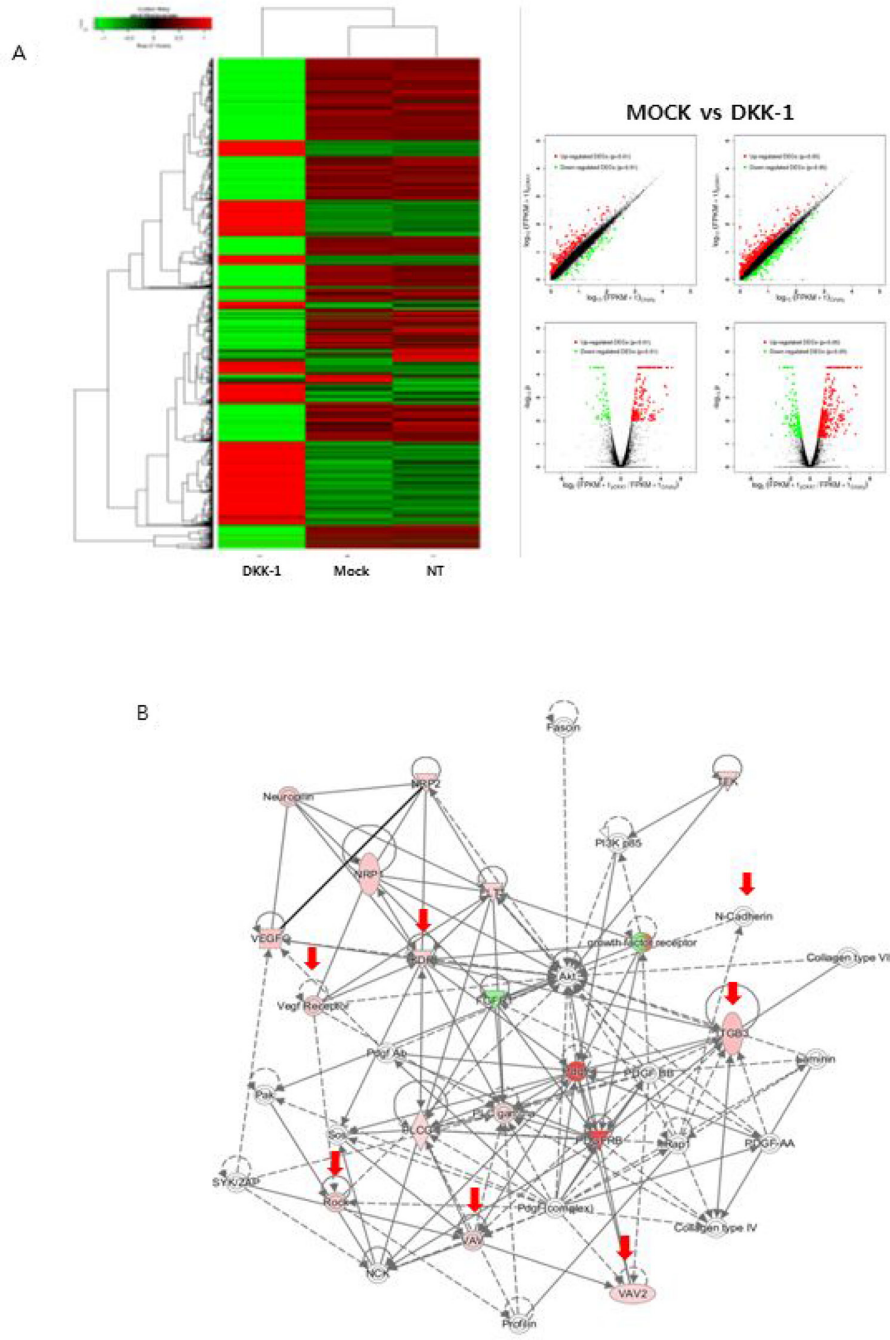
Effect of DKK-1 on gene expression DKK-1 stimulation influenced the expression of genes related to angiogenesis and cell morphology (**A**). A protein-protein interaction analysis indicated that VEGFR2 and N-cadherin are involved in the DKK1mediated enhancement of the EnMT potential of HUVECs (**B**).

DKK-1 stimulation using conditioned medium influenced the expression of genes related to angiogenesis and cell morphology (Figure 4A). A protein-protein interaction analysis indicated that the effect of DKK-1 stimulation on the EnMT potential of HUVECs is related to changes in the expression of several genes, including vascular endothelial growth factor receptor 2 (VEGFR2) and N-cadherin (Figure 4B). In contrast, the expression of genes related to cell proliferation and stability was not influenced by DKK-1 stimulation. Treatment with rDKK-1 yielded similar findings (Supplementary Figure 5). In addition to the high expression of VEGFR2 after DKK-1 stimulation, it has been known that VEGFR2 can promote angiogenesis and blockage of VEGFR2 can prohibit cancer invasion. Accordingly, we focused on VEGFR2 in our following experiments.

### DKK-1 induces angiogenesis via the VEGFR2 signaling cascade

**Figure 5:**
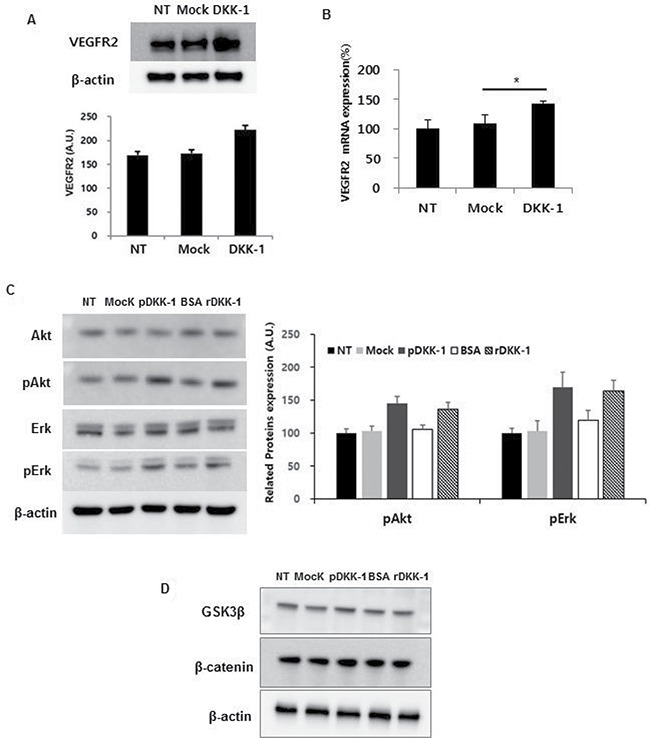
DKK-1 induces angiogenesis via the VEGFR2 signaling cascade Treatment of HUVECs with DKK-1 conditioned medium resulted in increased expression of VEGFR2, and phosphorylation of downstream molecules such as Akt and Erk, compared to the controls (**A–C**). However, β-catenin and GSK3β expression was unaffected (**D**).

The role of the VEGFR2 signaling cascade in DKK-1-induced angiogenesis was next evaluated. Simulation of HUVECs with DKK-1-conditioned medium resulted in increased expression of VEGFR2 compared to the controls (Figure 5A and 5B). Moreover, treatment with rDKK-1 induced increased expression of VEGFR2 in a concentration-dependent manner (Supplementary Figure 6). In addition, phosphorylation of VEGFR2 downstream molecules (e.g., Akt and Erk) was increased by DKK-1 stimulation (Figure 5C).

Because DKK-1 is an antagonist of the Wnt signaling pathway [10, 11], the expression of genes related to Wnt signaling in DKK-1-stimulated HUVECs was determined. No significant change in β-catenin or GSK3β expression was observed (Figure 5D). This suggests that the effect of DKK-1 stimulation on VEGFR2 expression is independent of the Wnt signaling pathway.

## DISCUSSION

**Figure 6:**
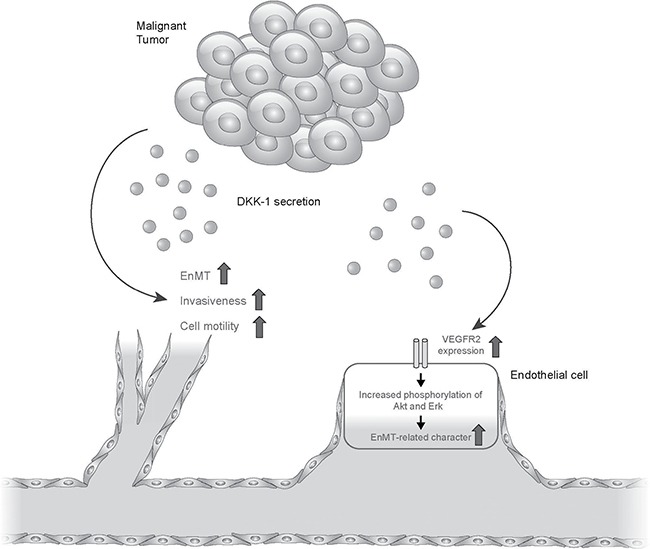
Conclusive figure summarizing the results

In this study, DKK-1 expression was high in HCC mice tissue and human HCC cell lines, in agreement with previous reports [9, 17], but it was not expressed in HUVECs. In addition, DKK-1 stimulation potentiated angiogenesis—as reflected by increased motility, invasion, and tube formation—by HUVEC cells. This was supported by increases in the expression of EnMT markers, such as N-cadherin, Twist and vimentin, and the decrease in VE-cadherin expression. The increased EnMT potential of DKK-1-stimulated HUVECs was found to be related to the activation of VEGFR2 and its downstream molecules, such as Akt and Erk. Finally, we found no significant change in β-catenin and GSK3β expression, indicating that DKK-1 stimulation influences angiogenesis by upregulating VEGFR2 expression (Figure 6).

A significant association between DKK-1 and angiogenesis has been reported previously. Tung *et al*. [13] observed that DKK-1-expressing tumors, established by injection of PLC/PRF/5 HCC cells into mice, showed an increased microvessel density score. Moreover, DKK-1 mediates endothelial cell activation by suppressing the Wnt/β-catenin pathway [14]. In this study, using an *in vivo* microvascular remodeling animal model, DKK-1 significantly increased vascular density and vessel diameter in rats. Similarly, Weng *et al*. [12] reported that DKK-1 promoted angiogenesis and cartilaginous degradation by synovial fibroblasts, accelerating synovial angiogenesis and cartilage destruction. Furthermore, DKK-1 treatment increased vessel formation in implanted Matrigel plugs and xenografted tumors [11]. Taken together, the above findings suggest that DKK-1 plays a role in microvascular remodeling and tumor angiogenesis.

However, conflicting results have also been reported. Inhibition of angiogenesis by DKK-1 was demonstrated due to its mitigation of ocular pathological neovascularization, characterized by excessive Wnt signaling [18]. Recently, it was reported that endothelial-cell-specific overexpression of DKK-1 in mice results in important vessel defects, including reductions in radial vessel expansion and the number of tip cells and filopodia in the retina [19]. The reasons for these discrepancies regarding the influence of DKK-1 on angiogenesis are unclear. Although further investigations are warranted, differences in the DKK-1 dose, method of DKK-1 manipulation, or functions of DKK-1 under various physiological conditions can be hypothesized.

Most cellular activities regulated by Wnt signaling are related to angiogenesis, particularly endothelial cell proliferation and morphogenesis. The role of Wnt in vascular development has been confirmed using targeted deletion mutants of Wnt signaling components in animal models [20, 21]. In addition, aberrant activation of Wnt signaling plays an important role in hepatocarcinogenesis. Consistently, inhibition of Wnt signaling exerted potent anti-HCC activity by increasing the rate of apoptosis of tumor cells and impairing tumor vascularization [22]. Given this role of Wnt signaling in vascular development, angiogenesis, and hepatocarcinogenesis, DKK-1 may be a good target for treating hypervascular tumors (such as HCC) by modulating angiogenesis. In contrast to low or no DKK-1 expression in normal cells or tissue as shown in this study, several HCC cell lines had high DKK-1 expression, and the serum level of DKK-1 in HCC patients is significantly associated with HCC staging, HCC recurrence after curative resection, and poor prognosis [9, 17]. These phenomena can indicate the DKK-1-mediated promotion of cancer growth by activation of tumor angiogenesis and support the rationale that DKK-1 can be a cancer-specific therapeutic target for HCC treatments. Indeed, sorafenib, the only approved molecular-targeted agent, which can suppress angiogenesis and tumor growth by inhibiting the Raf/MEK/ERK signaling pathway and receptor tyrosine kinases [23], prolongs the survival of patients with HCC. Interestingly, we found that the stimulation of HUVEC cells using the decreased concentration of DKK-1 in culture media by si-DKK1 treatment, led to the decreased expression of VEGFR2 (Supplementary Figure 7). This phenomenon can raise the necessities for further *in-vivo* studies which can provide the evidences that simultaneous suppression of VEGFR2 by blocking DKK-1 and sorafenib treatment can have synergistic effects on HCC treatment.

In our study, VEGFR2 expression was increased, and its downstream molecules—such as Akt and Erk—showed increased phosphorylation after DKK-1 stimulation. VEGFR2 is a receptor tyrosine kinase expressed predominantly in endothelial cells [24]. Activation of VEGFR2 can initiate multiple signaling pathways that orchestrate a variety of complex biological effects, such as endothelial cell maturation, vessel lumen formation, vascular permeability, vasodilation, angiogenesis, and arteriogenesis [25]. Thus, it can be hypothesized that high DKK-1 expression results in activation of VEGFR2 expression and increased sensitivity to VEGF in endothelial cells, which is significantly correlated with DKK-1 expression [26], during coordination of angiogenesis. The above results provide a rationale to support targeting of VEGFR2 using sorafenib, and show the potential of DKK-1 as a target of therapy [27]. Indeed, an anti-DKK-1 antibody suppressed HCC progression in a xenograft mouse model [13].

DKK-1 binds to the Wnt co-receptor LRP5/6, which inhibits the formation of a ternary receptor complex, resulting in blockade of β-catenin signaling [28]. In addition to LRP5/6, DKK-1 also binds to Kremen (Krm)-1/2 with high affinity to inhibit Wnt/β-catenin signaling [2]. However, in our study, no significant changes in β-catenin and GSK3β, the components of Wnt signaling, were observed. Although we did not identify the receptor for DKK-1 that mediates its influence on angiogenesis, our finding suggests that DKK-1 activates angiogenesis independently of the Wnt signaling pathway. A recent study reported that carbonic anhydrase IX (CA9), which modulates tumor-associated cell migration and invasion, interacts with DKK-1 [29]. Therefore, further work is required to identify the receptors or molecules associated with this role of DKK-1.

To date, the influence of DKK-1 on various cancer types has been controversial: DKK-1 was down-regulated in colon cancer [30, 31], whereas its expression is high in hepatoblastomas, Wilms’ tumor, and multiple myeloma [32, 33]. Because we investigated the association between HCC and angiogenesis in this study, we could not provide evidences to explain the reason why DKK-1 acts differently in various cancer types. Although we believe that the tumor heterogeneity among different cancers, even in the same tumor [34, 35], and the different experimental designs with different experimental materials among the previous studies might be related to these discordant results in part, further well-designed studies are warranted to elucidate this issue.

In conclusion, although the mechanisms of action of secreted DKK-1, and its physiological relevance to angiogenesis, are not fully understood, our data show that DKK-1 can induce angiogenesis by regulating VEGFR2 independent of the Wnt signaling pathway, and suggest that DKK-1 is a potential therapeutic target.

## MATERIALS AND METHODS

### Cell culture and transfection

Huh-7 (KCLB60104, Korean Cell Line Bank), Hep3B, HepG2 (KCLB88065, Korean Cell Line Bank), SNU449, and SNU475 cells were cultured at 37°C with 5% CO_2_ in Dulbecco's modified Eagle's medium (DMEM; Gibco, Grand Island, NY, USA), modified Eagle's Medium (MEM; Gibco), or RPMI-1640 (Gibco) supplemented with 10% fetal bovine serum (FBS; Gibco). SNU449 and SNU475 cells were derived from a primary HCC taken from a Korean patient, The 293 cells were cultured at 37°C with 5% CO_2_ in DMEM. HUVECs were cultured at 37°C with 5% CO_2_ in EGM-2 Bullet Kit (Lonza, Walkersville, MD, USA). Cells were transfected with pcDNA3-DKK-1 (pDKK-1) using Fugene HD transfection agent (Promega, Madison, WI, USA) according to the manufacturer's instructions. After transfection, the cells were incubated for 6 h and transferred to fresh culture medium.

### Cell growth assay

HCC cell growth rates were measured using the 3-(4,5-dimethylthiazol-2-yl)-2,5-diphenyltetrazolium bromide (MTT; Amresco, Solon, OH, USA) method. First, cells were seeded in a 96-well plate at 5 × 10^3^ cells/well and incubated at 37°C for 24 h in serum-free medium. Then, cells were transfected with pDKK-1 or rDKK-1as described above. After cultivation for 24 h, cells were transferred to culture medium containing 10% FBS. MTT was added to the wells, and the plates were incubated at 37°C for 3–4 hours to allow dye to penetrate the cells. The medium was removed and treated with dimethylsulfoxide (Sigma-Aldrich, St Louis, MO, USA) for 5 min at room temperature; its absorbance at 595 nm was analyzed using a spectrophotometer (Molecular Devices, Sunnyvale, CA, USA).

### mRNA isolation and real-time RT-PCR

RNA was extracted using a QIAGEN RNeasy mini kit (QIAGEN, Hilden, Germany) according to the manufacturer's instructions and reverse-transcribed (RT) using a Clontech RT Kit (Clontech, Mountain View, CA, USA). qPCR analysis was performed using SYBR Green Master Mix (Applied Biosystems, Foster City, CA, USA) and specific PCR primers (Supplementary Table 1). Amplification efficiencies were calculated for all primers using serial dilutions of pooled cDNA samples. The data were calculated, using the comparative (ΔΔCt) method, as the expression ratio to β-actin, the housekeeping gene. Data are shown as means ± standard error of the mean (SEM) of at least three independent experiments.

### Immunoblot analysis

The proteins were separated according to their molecular weight via sodium dodecyl sulfate-polyacrylamide gel electrophoresis (SDS-PAGE), transferred to a PVDF membrane (GE Healthcare, Amersham, UK), and probed with mouse monoclonal antibodies or rabbit polyclonal antibodies specific for the proteins of interest. The blots were developed using the enhanced chemiluminescence (ECL) technique (PerkinElmer, Boston, MA, USA) according to the manufacturer's instructions, and the level of each protein was quantified and compared (DKK-1, VE-cadherin and Vimentin: Abcam, Cambridge, UK; VEGFR2, β-actin, AKT, pAkt, ERK, pERK, GSK3b and N-cadherin: Cell Signaling, Danvers, MA, USA; Twist: Millipore, Billerica CA, USA). To detect secreted DKK-1, an enzyme-linked immunosorbent assay (ELISA; R&D Systems, Minneapolis, MN, USA) was performed according to the manufacturer's instructions.

### Angiogenesis assay

HUVEC invasiveness was assessed *in vitro* using a transwell chamber (Corning Costar, Cambridge, MA, USA). Then, 3 × 10^4^ HUVECS/well and treated rDKK-1 or conditioned medium of DKK-1–overexpressing 293 cells was transferred to each transwell chamber. Following incubation for 24 h, invading cells were stained with hematoxylin and eosin. The total number of invaded cells on the lower side of the filter was determined using a light microscope (Olympus America, Melville, NY, USA) at 40× magnification.

Tube formation by HUVECs was measured in Matrigel (BD Biosciences, San Jose, CA, USA). A 48-well plate (BD Falcon, Bedford, MA, USA) was coated with 50 μl Matrigel (10 mg/mL) and plated with HUVECs (7 × 10^4^/well); rDKK-1 or conditioned medium of DKK1overexpressing 293 cells was then added. The plates were incubated for 2.5 h, and tube formation was observed under a light microscope.

The migratory activity of DKK-1–stimulated HUVECs was assessed by migration assay. Cells were seeded at a density of 5 × 10^4^ cells/well onto 12-well plates and incubated for 24 h. A linear wound was made by scratching the monolayer with a sterile (white) pipette tip. After washing, cells were treated with 1 mL serum-free medium (control), rDKK-1 or conditioned medium of DKK-1–overexpressing 293 cells. Wound areas were photographed using an Olympus microscope (Olympus Optical Co., Tokyo, Japan).

### mRNA sequencing and genome mapping of sequence reads

Isolated total RNA was used to prepare an mRNA sequencing library using the Illumina TruSeq Stranded mRNA Sample Preparation kit (Illumina, San Diego, CA, USA) according to the manufacturer's protocol to generate single 8 bp indices for multiplexing. Library quality and size were assessed using an Agilent 2100 Bioanalyzer DNA kit (Agilent, Palo Alto, CA, USA). Potentially existing sequencing adapters and low-quality bases in the raw reads were trimmed using Cutadapt software. The cleaned high-quality reads were mapped to the human reference genome hg19 of the UCSC genome (https://genome.ucsc.edu) using TopHat2 software [36]. The sequencing libraries were prepared in a strand-specific manner using the Illumina strand-specific library preparation kit during mapping. To quantify the mapped reads on the human reference genome in terms of gene expression values, Cufflinks software with the strand-specific library option was used [37]. Gene annotation for the human reference genome hg19 in GTF format was used to quantify gene expression. Gene expression values were calculated in fragments per kilobase of transcript per million fragments mapped (FPKM). Differentially expressed genes between two biological conditions were analyzed using the Cuffdiff software in the Cufflinks package [37].

### Statistical analysis

Results were expressed as means ± SEM or frequencies (%). An independent *t*-test was performed to compare means between the control and experimental groups. All statistical analyses were performed using SPSS software (ver. 12.0; SPSS Inc., Chicago, IL, USA). A *p*-value of < 0.05 was considered to indicate statistical significance.

## SUPPLEMENTARY MATERIALS FIGURES AND TABLE



## Abbreviations

DKK-1: dickkopf-1
HCC: hepatocellular carcinoma
EnMT: endothelial–mesenchymal transition
HUVECs: human umbilical vein endothelial cells
pDKK-1: pcDNA3-DKK-1
rDKK-1: recombinant DKK-1
pDKK-1: a high concentration of secreted DKK-1 protein
VEGFR2: vascular endothelial growth factor receptor 2.
