# Supplementary material for: Dickkopf-1 induces angiogenesis via VEGF receptor 2 regulation independent of the Wnt signaling pathway
Source: Oncotarget. 2017 Aug 1;8(35):58974–84. doi: 10.18632/oncotarget.19769 (PMC5601707; doi:10.18632/oncotarget.19769)
Supplement: Supplementary file 1 [file oncotarget-08-58974-s001.pdf]

Dickkopf-1 induces angiogenesis via VEGF receptor 2 regulation independent of the Wnt signaling pathway

SUPPLEMENTARY MATERIALS

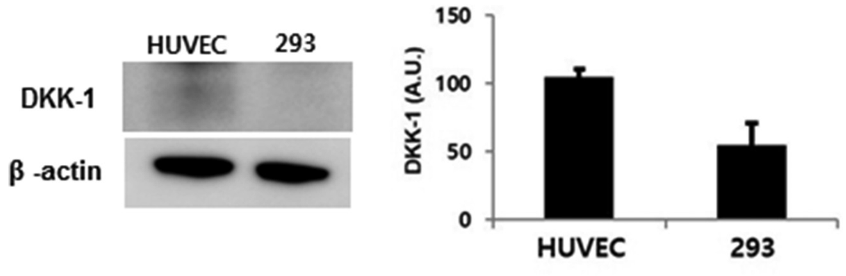

Supplementary Figure 1: DKK-1 was not expressed in HUVECs or 293 cells.

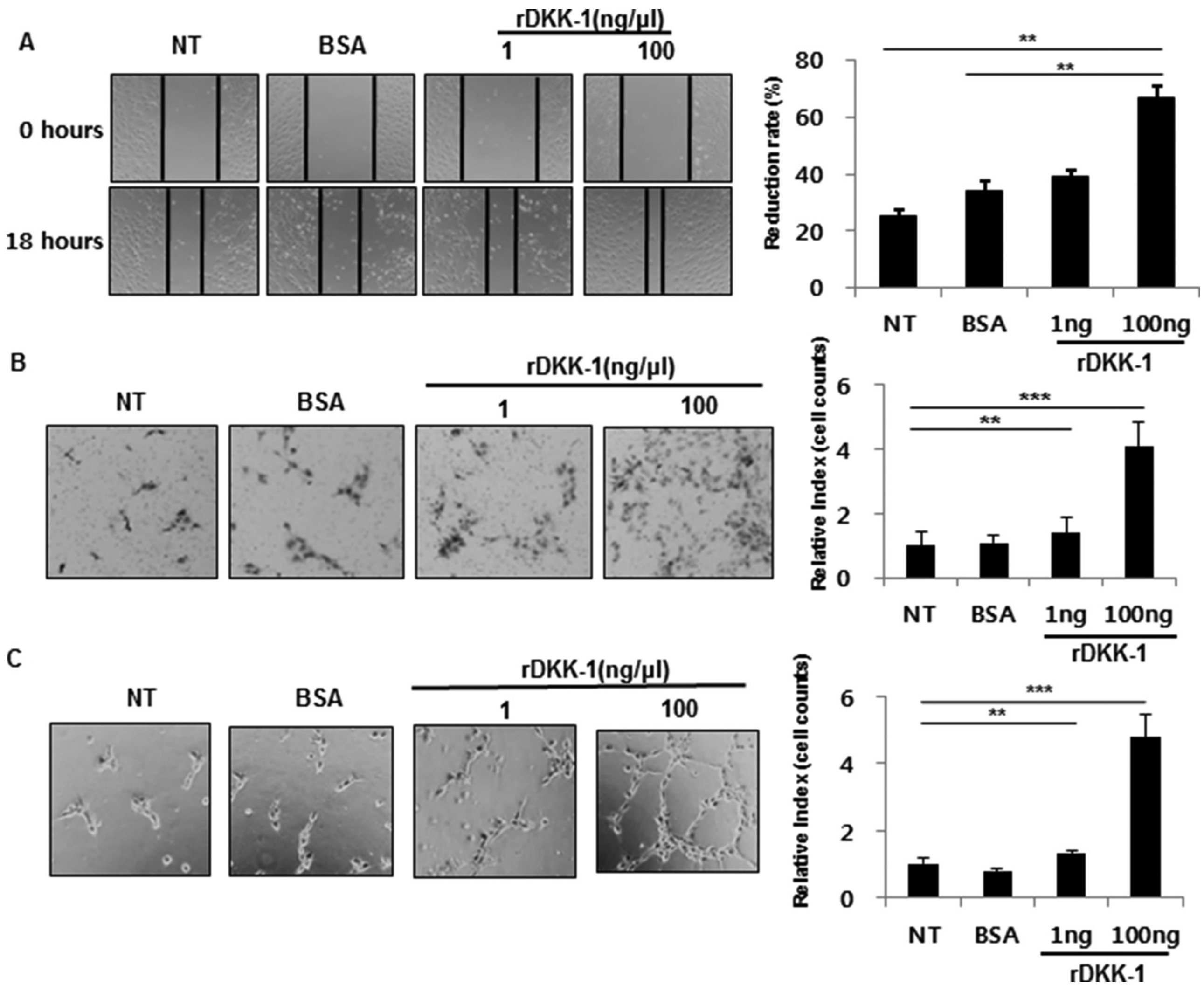

Supplementary Figure 2: Recombinant DKK-1 (rDKK-1) increases the motility, invasion, and tube formation of HUVECs. Treatment with rDKK-1 led to concentration-dependent increases in motility, invasion, and tube formation of HUVECs. NT, non-treated; BSA, Bovine serum albumin.

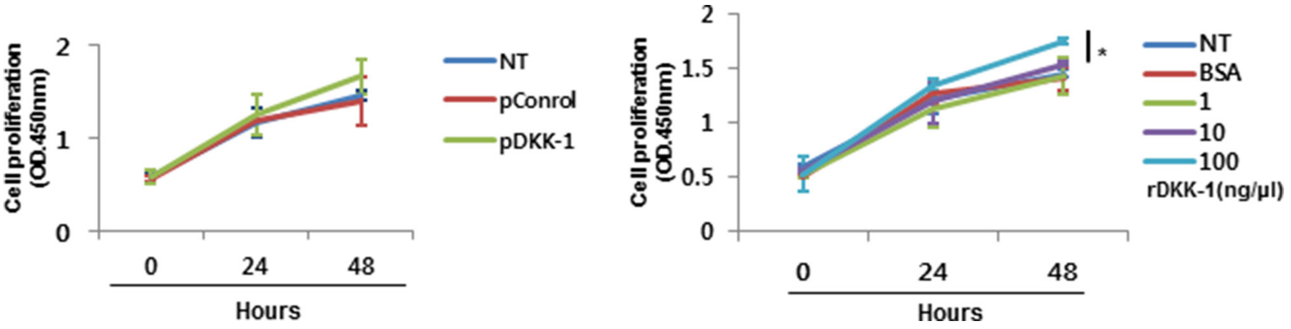

Supplementary Figure 3: Proliferation of HUVECs. DKK-1 did not affect the proliferation of HUVECs. NT, non-treated; BSA, Bovine serum albumin.

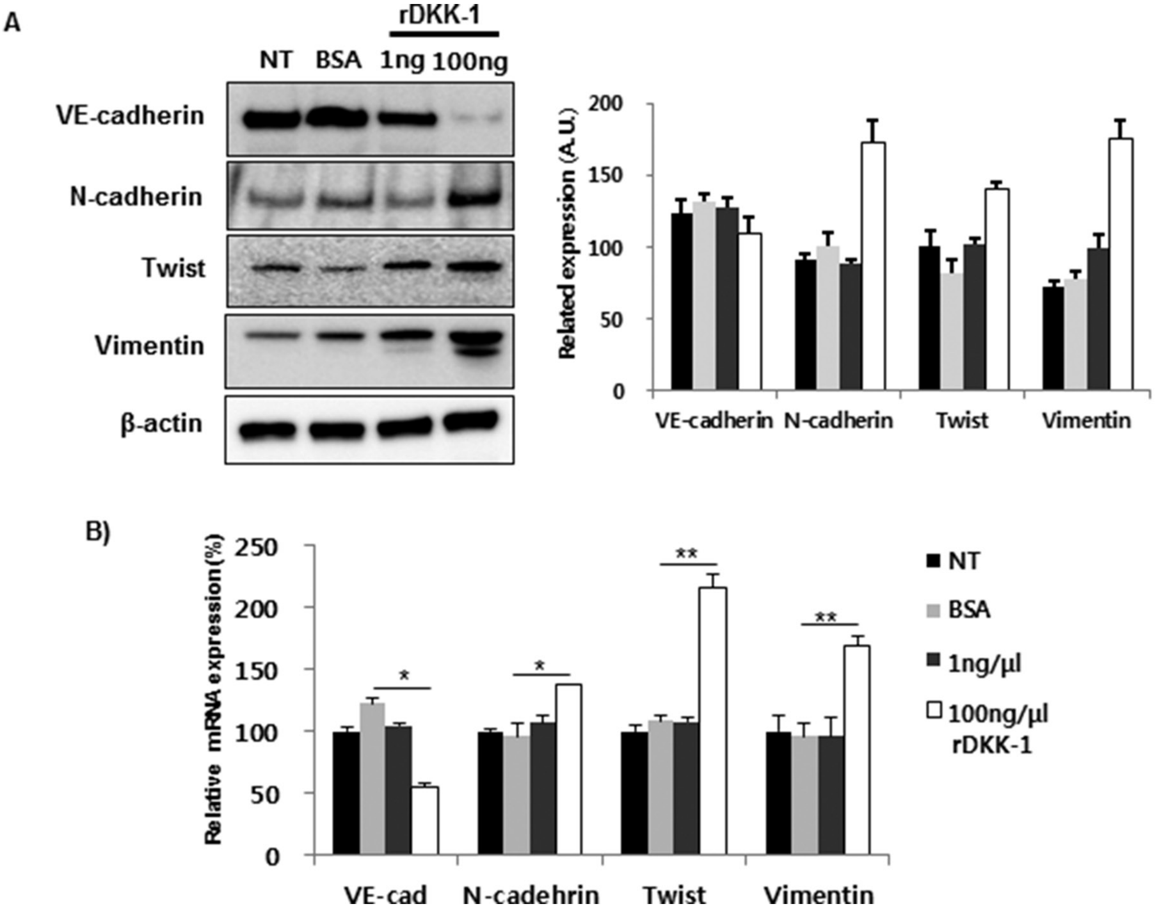

Supplementary Figure 4: rDKK-1 increased the expression of EnMT markers in HUVECs. rDKK-1 stimulation resulted in increased N-cadherin, Twist and vimentin expression, and decreased VE-cadherin expression, compared to the controls by western blot (**A**) and mRNA level (**B**). NT, non-treated; BSA, Bovine serum albumin.

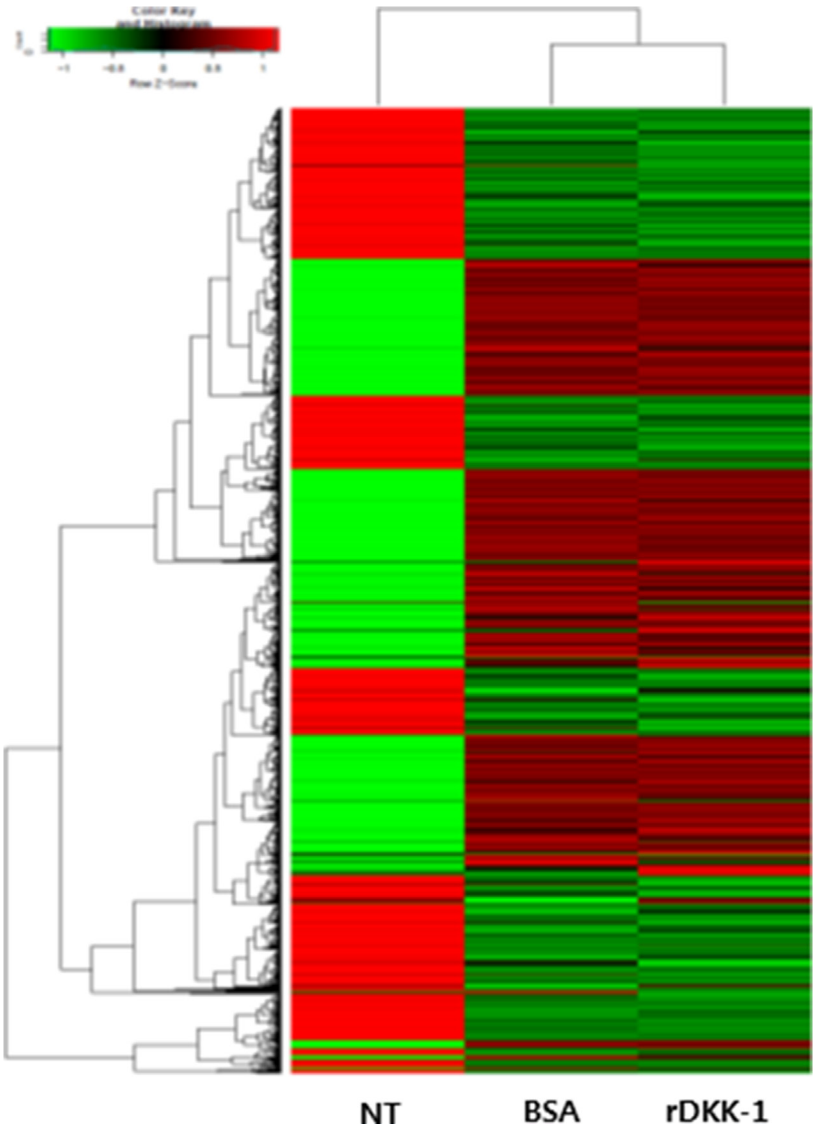

Supplementary Figure 5: Effect of rDKK-1 on gene expression profile. Treatment with rDKK-1 affected the expression of genes related to angiogenesis and cell morphology (**A**). A protein-protein interaction analysis indicated that VEGFR2 and N-cadherin are involved in the DKK-1-mediated increased EnMT potential of HUVECs (**B**). NT, non-treated; BSA, Bovine serum albumin.

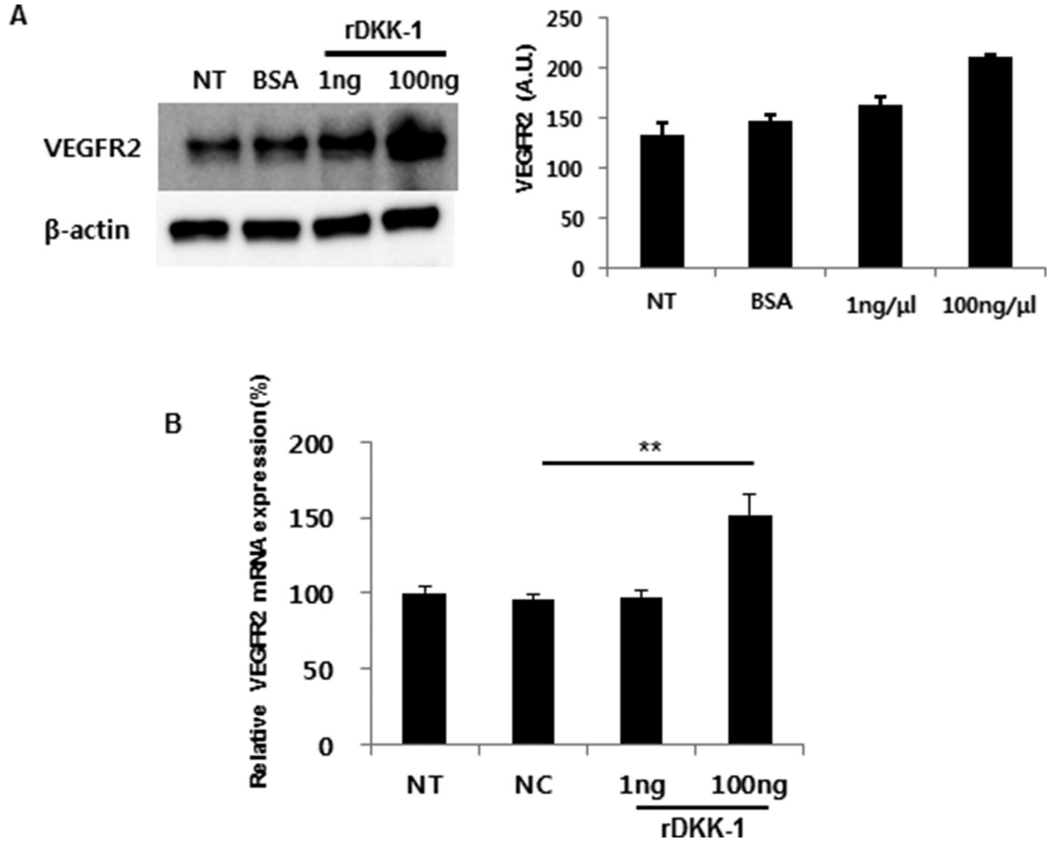

Supplementary Figure 6: rDKK-1 induces angiogenesis via VEGFR2. Treatment of HUVECs with DKK-1 conditioned medium resulted in a concentration dependent increase in VEGFR2 expression compared to the controls. NT, non-treated; BSA, Bovine serum albumin.

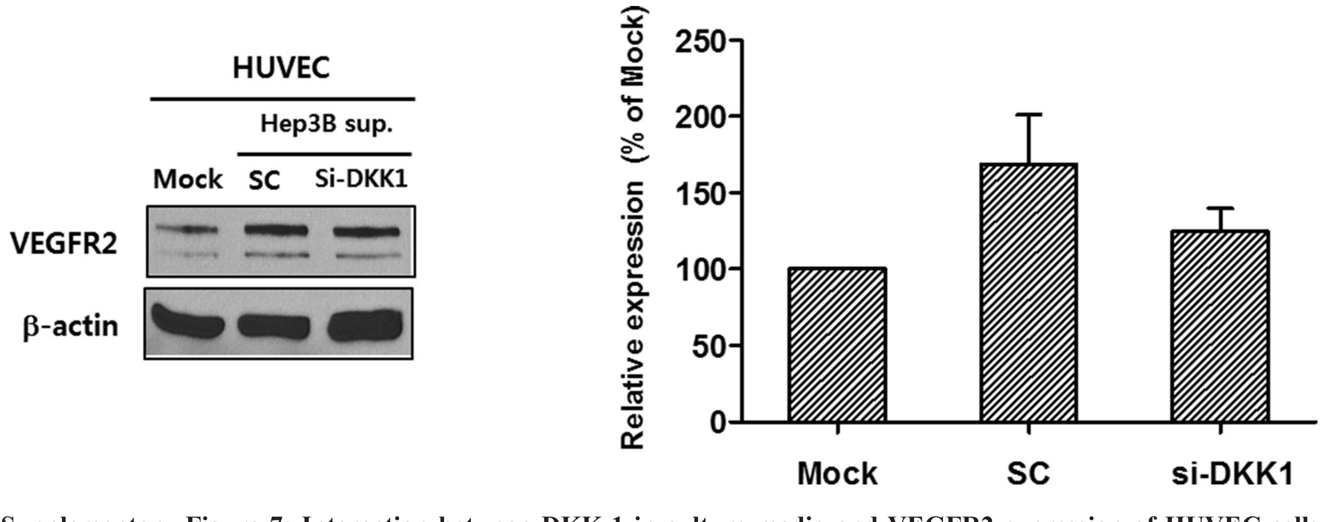

Supplementary Figure 7: Interaction between DKK-1 in culture media and VEGFR2 expression of HUVEC cells. The stimulation of HUVEC cells using decreased concentration of DKK-1 in culture media by si-DKK1 treatment, led to the decreased expression of VEGFR2.

Supplementary Table 1: Information of real time quantitative PCR primers

	Forward	Reverse
**DKK-1**	GTTACTGTGGAGAAGGTCTGTC	GTTCACTGCATTGGATAGCTG
**hVEGFR2**	GAGGATCTTGAGTTCAGACATGAG	TTGGAATTGACAAGACAGCAAC
**hVE-cadherin**	GCCAAGTACTTATTTCCTCGGT	GAGGAGCTCACTGTGGATTC
**hN-cadherin**	CATACCACAAACATCAGCACAAG	GTTTGCCAGTGTGACTCCA
**hTwist**	CTGCCCTCGGACAAGCTG	CTAGTGGGACGCGGACAT
**hVimentin**	CTTGAACGGAAAGTGGAA	GTCAGGCTTGGAAACGTC
